# Multicomponent MR Image Denoising

**DOI:** 10.1155/2009/756897

**Published:** 2009-10-29

**Authors:** José V. Manjón, Neil A. Thacker, Juan J. Lull, Gracian Garcia-Martí, Luís Martí-Bonmatí, Montserrat Robles

**Affiliations:** ^1^Instituto de Aplicaciones de las Tecnologías de la Información y de las Comunicaciones Avanzadas (ITACA), Universidad Politécnica de Valencia, Camino de Vera s/n, 46022 Valencia, Spain; ^2^Imaging Science and Biomedical Engineering Division, Medical School, University of Manchester, Stopford Building, Oxford Road, Manchester M13 9PT, UK; ^3^Department of Radiology, Quirón Hospital, Blasco Ibáñez, 14, 46010 Valencia, Spain

## Abstract

Magnetic Resonance images are normally corrupted by random noise from the measurement process complicating the automatic feature extraction and analysis of clinical data. It is because of this reason that denoising methods have been traditionally applied to improve MR image quality. Many of these methods use the information of a single image without taking into consideration the intrinsic multicomponent nature of MR images. In this paper we propose a new filter to reduce random noise in multicomponent MR images by spatially averaging similar pixels using information from all available image components to perform the denoising process. The proposed algorithm also uses a local Principal Component Analysis decomposition as a postprocessing step to remove more noise by using information not only in the spatial domain but also in the intercomponent domain dealing in a higher noise reduction without significantly affecting the original image resolution. The proposed method has been compared with similar state-of-art methods over synthetic and real clinical multicomponent MR images showing an improved performance in all cases analyzed.

## 1. Introduction

Image denoising is a common preprocessing step in many Magnetic Resonance (MR) image processing and analysis tasks, such as segmentation [1], registration [2] or parametric image synthesis [3]. Many filtering methods use the signal averaging principle which is based on the natural spatial pattern redundancy in the images. In this sense, Gaussian filters have been largely used in some applications such as functional MR imaging (fMRI) [4]. However, they have the disadvantage of blurring edges due to the averaging of nonsimilar patterns.

 In order to avoid this problem, many edge preserving filters have been proposed. Probably the most well-known filter is the Anisotropic Diffusion Filter (ADF) [5, 6]. ADF respects edges by averaging pixels in the orthogonal direction of the local gradient. However, such filtering usually erases small features and transforms image statistics due to its edge enhancement effect resulting in unnatural images.

 Modern wavelet-based filters have also been applied to MR image denoising [7, 8]. Such filters, although effective, are prone to introduce characteristic artifacts (small spots) that can hamper the image analysis process.

 Many existing filters used in MRI work using a single image component or volume without taking into consideration the multicomponent intrinsic nature of MR studies. A typical MR study is comprised by many different types of images of the same patient (e.g., T1,T2, FLAIR, etc.), where after a registration process each voxel can be seen as vector with as many components as image types in the study.

 There are few methods that use this multicomponent information as a basis for the denoising process. One of the first attempts to use this information was the multicomponent ADF proposed by Gerig et al. [6]. In this method, the authors proposed using the gradient information of different image sequences of the same subject to constrain the diffusion process.

 In the context of wavelet thresholding, a new denoising technique for multicomponent images, exploiting interscale and intercomponent correlations was recently proposed by Scheunders and Backer [9]. This technique was demonstrated to outperform similar single and multicomponent wavelet thresholding techniques.

 On the other hand, a partial volume modelling-based approach has been also recently proposed by Thacker and Pokric [10] where the filtering was performed using multidimensional data and a partial volume data density model. This approach abandons altogether local smoothness constraints and achieves noise reduction by enforcing agreement between measured data using underlying tissue proportions computed from a physics-based image formation model.

 In the present work, a novel method for multicomponent MR image denoising is presented. This method is inspired on a new filter recently proposed by Buades et al. [11] known as Nonlocal Means (NLM). The main hypothesis in this work is that when multiple MR images of different type or different acquisition times are available, the filtering process can be improved by using the additional correlated information in such images. The proposed method will work on both spatial and intercomponent domain. In the next sections, the proposed filter is fully described and its application in real and simulated multicomponent MR data is evaluated and compared with related previous state-of-the art methods.

## 2. Material and Methods

The NLM filter is a neighbourhood filter [12] which achieves denoising by averaging similar image pixels according to their intensity similarity. The main difference between the NLM and previous related filters is that the similarity between pixels has been made more robust to the noise level by using region comparison rather than pixel comparison; furthermore, pattern redundancy has been not restricted to be local (nonlocal). That is, pixels far from the pixel being filtered are not penalized due to its distance to the current pixel, as for example, happens in the bilateral filter [13].

### 2.1. NLM Filter

Given an image *Y*, the filtered value at pixel *p* using the NLM method is computed as a weighted average of all the pixels in search area Ω within the image:

(1) $$\begin{array}{c} \text{NLM}\left(Y\left(p\right)\right)=\underset{\forall q\in \Omega }{\sum }w\left(N_{p},N_{q}\right)Y\left(q\right),\\ 0\leq w\left(p,q\right)\leq 1\;\;\underset{\forall q\in \Omega }{\sum }w\left(N_{p},N_{q}\right)=1, \end{array}$$

where *Y*(*p*) is the pixel being filtered and *Y*(*q*) represents each one of the pixels in the image. The weights *w*(*N* *p*, *N* *q*) are based on the similarity between the neighbourhoods *N_p_* and *N_q_* of pixels *Y*(*p*) and *Y*(*q*). The neighbourhood *N_i_* is defined as a square window centered on pixel *i* with a user-defined radius *R*_sim_. The region Ω is defined as a squared region surrounding the pixel being processed of radius *R*_search_. Although the original method claims that this region Ω can be the entire image due to computational reasons this region has to be restricted to be of smaller size.

 The similarity *w*(*N* *p*, *N* *q*) is calculated as

(2) $$\begin{array}{c} w\left(N_{p},N_{q}\right)=\frac{1}{Z\left(p\right)}e^{-d(N_{p},N_{q})/h^{2}},\\ Z\left(p\right)=\underset{\forall q\in Y}{\sum }e^{-d(N_{p},N_{q})/h^{2}}, \end{array}$$

Where *Z*(*p*) is the normalizing constant, *h* is a exponential decay control parameter and *d* is a Gaussian weighted squared Euclidian distance (with standard deviation 1) of all the pixels of each neighbourhood as defined in [11]:

(3) $$d\left(N_{p},N_{q}\right)={\left\|\left(Y\left(N_{p}\right)-Y\left(N_{q}\right)\right)\right\|}_{R_{\text{sim}}}^{2}.$$

This measure penalizes pixels far from the center of the neighbourhood window giving more weight to pixels near the center of the window in the distance computation.

 In (2) there is a special case when *p* = *q*. As the self distance is zero, it can produce an over-weighting effect. To solve this problem, Buades proposed to calculate *d*(*N* *p*, *N* *p*) as the minimum distance of the rest of the pixels in the image [14]:

(4) $$d\left(N_{p},N_{p}\right)=\;\min\;\left(d\left(N_{p},N_{q}\right),\;\;\forall q\neq p\right).$$

**Figure 1:**
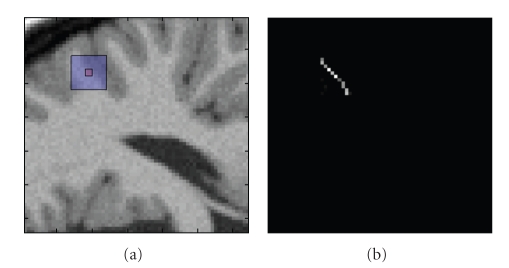
(a) Each filtered pixel in the image (e.g., center pixel of the red square) is computed as the weighted average of all the pixels of the search window (blue square). (b) Weights of pixels of the search region as calculated by (2). As can be observed in the example edge pixels are averaged with other edge pixels preventing the blurring effect.

In Figure 1, an example of the NLM estimated weights for a small squared region is displayed. As can be noticed, the NLM method finds successfully as similar to the current pixel (small red square) other edge pixels within the search window.

### 2.2. Multicomponent Nonlocal Means (MNLM)

As in medical MR imaging the acquisition of multiple images with different acquisition parameters is a common practice, the above method can be extended to be used in a multicomponent framework. Effectively, the similarity measure can be better estimated by combining information not only from the surrounding pixels but also using information of the different components in a similar manner as performed on colour image denoising using the RGB space [12].

 Therefore, the multicomponent similarity function is computed as follows:

(5) $$w\left(N_{p},N_{q}\right)=\frac{1}{Z\left(p\right)}e^{-(\sum_{i=1}^{C}(d(N_{p}^{i},N_{q}^{i})/{h^{i}}^{2})/C)},$$

where

(6) $$Z\left(p\right)=\underset{\forall q}{\sum }e^{-(\sum_{i=1}^{C}(d(N_{p}^{i},N_{q}^{i})/{h^{i}}^{2})/C)},$$

Where *C* is the number of components and *h^i^* parameter is related with the noise standard deviation of each image.

 The MNLM algorithm, as in the single component case, has three free parameters, and the filtering results highly depend on their correct setting.

 The first parameter, *R*_search_, is the radius of the search window. Although the original method claimed to use all the pixels in the image by taking the weighted average of every pixel, this is inefficient if the only similar locations are relatively nearby. Besides, the computational burden would be prohibitive for clinical applications. Therefore, the search window has to be reduced to a local window Ω of smaller size.

 The second parameter, *R*_sim_, is the radius of the neighbourhood window used to compute the similarity between two pixels. If the value of *R*_sim_ is increased the similarity measure will be more robust but fewer similar neighbourhoods will be found.

 The third parameter, *h*, is related to the decay of the exponential curve and controls the degree of smoothing. If *h* is too small, little noise will be removed while if *h* is set too high, the image will become blurry.

 In our experiments, *R*_search_ was set to 10 (this is a 21 × 21 search window), which has been found experimentally as a good compromise between noise reduction and computational burden. For 2D processing an *R*_sim_ of 2 has been found to be a good choice for typical noise levels in MR imaging [15]. However, for multicomponent imaging the additional information from other components allows the use of a smaller similarity region (in our experiments an *R*_sim_ = 1 was used, i.e., a 3 × 3 similarity region) dealing in a more point specific similarity measure and therefore increasing the number of similar patches. Finally, the parameter h_*i*_ was set to $\sigma_{i}\sqrt{2}$, being *σ*_*i*_ the noise standard deviation in each image component (this value was found to be experimentally the best choice).

### 2.3. Proposed Method

Although the MNLM filter obtains remarkable results, a number of optimizations can be done to increase its accuracy.

#### 2.3.1. Similarity Function

In (3) the distance between two equal noisy patches will have an average distance equal to 2*σ* ^2^ [11]. Therefore, its associated weight will not be equal to 1 as expected (assuming *h* ^2^ = 2*σ* ^2^) but 1/e (see (2) and (5)). This can be easily solved by simply subtracting 1 from exponent in the weight computation; so similar pixels will have a weight close to 1 (this has the same effect than subtracting 2*σ* ^2^ to distance as calculated in (3)). To avoid negative values due to the subtraction operation, we calculate the normalized distance as the maximum of the distance after the subtraction and 0. This optimization is specially effective on low-noise conditions:

(7) $$w\left(N_{p},N_{q}\right)=\frac{1}{Z\left(p\right)}e^{-(\max\;((\sum_{i=1}^{C}d(N_{p}^{i},N_{q}^{i})/{h^{i}}^{2}/C)-1,0))}.$$

In this case, the multicomponent similarity function is computed using the same definition as in (5) but subtracting 1 to averaged distance to obtain a weight close to 1 when computing the distance of two equal patches.

#### 2.3.2. Multicomponent Preselection

Another useful improvement is to perform a pixel preselection in order to save useless computations and to improve filtering results by excluding nonsimilar pixels in the averaging process. Several methods of preselection have been already proposed using gradient information [16] or local image moments [17].

 In the present work, we propose selection of those pixels with a difference of their first local moment (mean value of a 3 × 3 image patch) smaller than the *k* *σ*_i_/$\sqrt{n}$ (being *σ*_*i*_ the noise standard deviation in the image *i* and *n* the number of pixels used to compute the mean) in each image:

(8) $$w\left(N_{p},N_{q}\right)=\{\begin{array}{cc} \frac{1}{Z\left(p\right)}e^{-d{\left(p,q\right)}^{*}}, & \text{if}\;\left(\left|\mu_{pi}-\mu_{qi}\right|<k\sigma_{i}/\sqrt{n}\right),\\ & \forall i\in \left[1,C\right],\\ 0, & \text{otherwise}, \end{array}$$

where

(9) $$d{\left(p,q\right)}^{*}=\max\;\left(\frac{\sum_{i=1}^{C}d\left(N_{p}^{i},N_{q}^{i}\right)/{h^{i}}^{2}}{C}-1,0\right).$$

In single image denoising the value of *k* can be set to 3 which correspond to the third quantile of a standard normal distribution. Patches with mean value differences higher than this threshold have a very small probability to be similar to the current patch. In multicomponent data this threshold was found experimentally to be too restrictive. In our experiments, we have set *k* = 4 which was found to be a better option. Experiments using the local variance were performed to improve the preselection step but no significant improvements were found. We will refer to the optimized MNLM method including optimizations 1 and 2 as OMNLM.

#### 2.3.3. Principal Component Analysis (PCA) Denoising

In the presented method, noise reduction is achieved by averaging similar pixels in the spatial domain. However, a higher noise reduction can be obtained by using information in the intercomponent domain as well.

 Principal Component Analysis and related approaches have been previously used to reduce noise in the images [18, 19]. In this context, noise removal is done by decomposing the signal into the local principal components, attenuating less relevant components and reconstructing again.

 The simplest approach for multicomponent image denoising is to perform PCA decomposition of the image series and to remove all the components that have a variance similar to the image noise. However, although effective, this approach requires the number of images to be higher than the number of significant components of the image. Besides, image inhomogeneities typically present in MRI make it more difficult to obtain noise-related components due to a higher number of significant components required to represent the data. This limits the applicability of the technique to studies that acquire large image series such us fMRI [20]. This problem can be overcome by performing PCA decomposition over small local windows instead of the whole image [18].

 The basic idea is that every pixel of the image can be denoised by decomposing the local surrounding square window of a given radius for the different images in the corresponding components and attenuating the less significant ones. In the proposed approach for each pixel, the PCA decomposition of a local matrix of *N* × *K* data is calculated, *N* being the number of pixels of the local window (in our experiments, *N* = 9 was used, i.e., a 3 × 3 local window) and *K* the number of components. The obtained components are processed (using hard or soft thresholding) before recomposing the original matrix. This means that, for example, a local window containing a single tissue can be well approximated by its mean value and therefore all components can be suppressed dealing in a much stronger noise reduction than in the global case. Finally, after reconstruction the filtered intensity value for a particular pixel can be obtained by averaging estimates of multiple overlapping windows. Such averaging allows removal of more noise, in a similar way to the translation invariant denoising proposed by Coifman and Donoho [21].

 The attenuation can be done using hard or soft thresholding similar to wavelet-based denoising. In our case, we use soft thresholding where each component is multiplied by a factor *f* defined as.

(10) $$f=\frac{1}{{\left(1+e^{-(\sigma_{\text{component}}-2\sigma_{\text{noise}})}\right)}^{2}}.$$

This process is applied as a postprocessing step after the application of the OMNLM filter to reduce noise in the images by using information of the spatial domain as well as in the intercomponent domain. We will refer to the combination of OMNLM method and PCA postprocessing as OMNLM-PCA.

 The proposed method can be summarized as follows.

1. *Spatial denoising*, apply the OMNLM to the noisy data to reduce noise in the spatial domain.
2. *PCA postprocessing*, perform a final Local PCA denoising over the spatially filtered images using an estimate of the local remaining noise.

## 3. Experiments and Results

**Figure 2:**
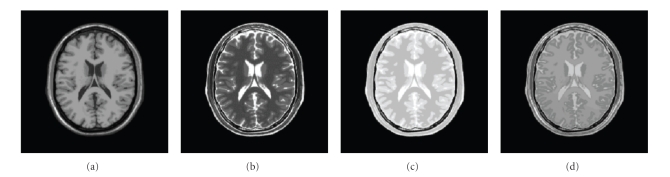
From left to right: synthetic noise free T1, T2, and PD (low and high flip angle) weighted MR images.

To evaluate the proposed method over synthetic cases, a slice of simulated T1, T2, and two PD (low and high flip angles) weighted images (1 mm^3^ voxel resolution and 8 bit quantization) from the BrainWeb phantom was used [22] (see Figure 2). Noise in MRI can be Gaussian or Rician distributed [23] depending on the image type (real or magnitude data). In our experiments, all images were corrupted with Gaussian-distributed random noise with different levels since at brain tissues noise distribution can be generally well approximated by a Gaussian distribution. As our main interest is to remove noise from the region of interest (typically the brain), in our experiments all the measures have been obtained from a region corresponding to the brain parenchyma since noise removal at background can bias the measures.

 The accuracy of the proposed filter (OMNLM and OMNLM-PCA versions) over usual noise levels (1 to 9% of the maximum T1-weighted image intensity) was evaluated and compared with the MNLM method. Also, experiments were performed to evaluate the effect of adding multiple images in the image denoising process. All experiments were performed using MATLAB 7.0 (MathWorks Inc., Natick, USA).

To measure the accuracy of the different compared approaches the Root Mean Squared Error (RMSE) and Multicomponent Root Mean Squared Error (MRMSE) were used:

(11) $$\begin{array}{c} \text{RMSE}=\sqrt{\frac{1}{M}\overset{M}{\underset{i=1}{\sum }}{\left(X(i)-F(i)\right)}^{2}},\\ \text{MRMSE}=\sqrt{\frac{1}{\text{CM}}\overset{C}{\underset{j=1}{\sum }}\overset{M}{\underset{i=1}{\sum }}{\left(X(i,j)-F(i,j)\right)}^{2}}, \end{array}$$

where *X*(*j*) is the *j*th noise free image component and *F*(*j*) is its denoised image version, both containing *M* pixels. *C* is the number of components. This measures was calculated only from data within a mask comprising the brain tissues.

 In Table 1, a comparison of the basic MNLM filter and the proposed OMNLM and OMNLM-PCA methods is presented. It can be noticed that the proposed optimizations improved significantly the accuracy of the basic filter for all the noise levels. The OMNLM-PCA version achieved the better results for all noise levels.

To evaluate the effect of adding new images to the filtering process only the RMSE of the T1-weighted image was measured when using different number of images for the pixel similarity estimation. It is clear that no PCA step was used when using a single image. Results comparing the basic MNLM filter and the proposed OMNLM-PCA filter can be observed in Table 2. Curiously, in the basic MNLM filter, the best results were obtained when using only two images (T1 and T2), probably because these images present good contrast between different tissues. In this case, the inclusion of the two PD images in the similarity measurements, did not improve the results but made them slightly worse behaving as a confounding factor.

Conversely, the proposed method presented a consistent improvement of the results as the number of images was increased. This was found to be mainly due to the preselection optimization which avoids averaging the current pixel with nonsimilar pixels.

### 3.1. Comparison with Other Multicomponent Denoising Methods

The described method was compared with the state-of-the art multicomponent denoising methods. Specifically, the compared methods were the multidimensional partial volume modelling method (MPVM) proposed by Thacker et al. [10] and the multicomponent method based on wavelet thresholding using Gaussian Mixture Modelling (MGMM) recently proposed by Scheunders and Backer [9]. These methods were applied with their default parameters (*sym4* wavelet type and depth equal 3 for the MGMM method and a 6 tissue model for the MPVM method). Results can be compared in Table 3.

**Figure 3:**
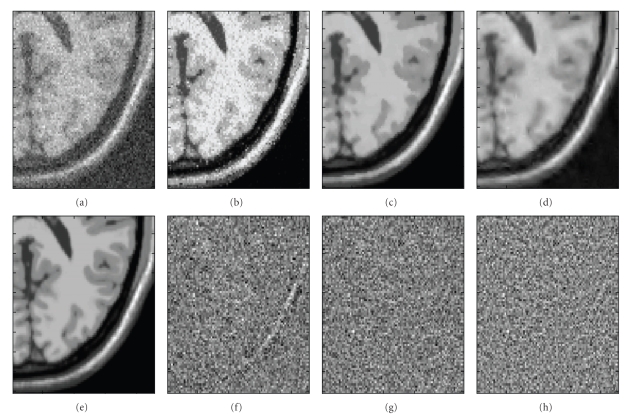
Detail of filtering results. (a) Noisy T1 image. (b) MPVM filter result. (c) OMNLM-PCA filter result. (d) MGGM filter result. (e) Noise Free T1 image. (f) MPVM residuals. (g) OMNLM-PCA residuals. (h) MGGM residuals. As can be noticed, almost no anatomical information is visible in the image residuals for the compared methods (except for MPVM). However, with the proposed method, the image details are better preserved than the others.

The best results were obtained by the proposed method for all noise levels. The MGMM method showed good results for low-noise levels while for medium and high noise levels this method tends to blur the images (Figure 3). The MPVM method was less accurate than the other methods. The execution times of the compared methods were 10 seconds for the MVPM method, 6 seconds for MGMM method and 33 seconds for the OMNLM-PCA method.

### 3.2. Comparison on Real Clinical Images

**Figure 4:**
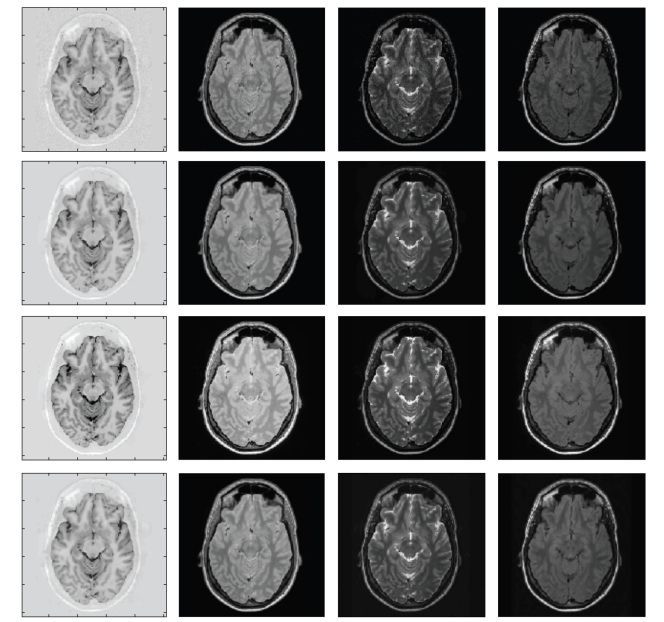
Denoising results (from top to down: original noisy images, denoised images with the OMNLM-PCA, MPVM, and MGMM methods).

To evaluate effectiveness of the different multicomponent approaches on real image conditions, four real clinical images were used (IRTSE, PD, T2, and FLAIR) (Figure 4). All images were acquired with a 1.5-T system (ACS-NT, with PowerTrack 6000 gradient subsystem; Phillips Medical Systems, Hamburg, Germany) with a birdcage head coil receiver. Data was in contiguous 3 mm thick sections throughout the brain, with an in-plane resolution of 0.89 mm (matrix, 256 × 204, field of view, 230 × 184 mm). All images were registered with in-house software developed in our lab. The noise level in each image was estimated using the LNE technique [10] which is based on the area of the probability distribution *p* of the second-order derivatives of the image (see (12)):

(12) $$\sigma_{\text{noise}}=\frac{\left(d\sum_{i=-d}^{d}p\left(i\right)\right)}{0.63}\;\text{where}\;d=\arg\;\min\;\left(\overset{d}{\underset{i=-d}{\sum }}p\left(i\right)-0.63\right).$$

To evaluate the efficiency of the different filters, two measures were used. One based on the image residuals (i.e., difference between the noisy and denoised images) and the other based on an estimation of the noise level after filtering (in this case a noise free image is not available for direct comparison). These measures were estimated from all the pixels belonging to the intracranial cavity as in this area the random noise can be approximately considered as Gaussian-distributed.

 The first measure was the Remaining Noise Fraction (RNF):

(13) $$\text{RNF}=\frac{\sigma_{\text{denoised}}}{\sigma_{\text{original}}},$$

where *σ*_denoised_ is the standard deviation of the noise after filtering and *σ*_original_ is the standard deviation of the noise in the original noisy image. Both standard deviations were estimated using the LNE technique.

 The second measure was obtained from the image residuals. This measure was the Number of Outliers in the residuals, defined as the number of pixels with values lying 3 times beyond the standard deviation of the image residuals (for Gaussian-distributed noise this is expected to be around the 0.05% of the data).

 The first measure relates with the amount of noise removed while the second is devised to measure the quality of the filtering process. Table 4 summarizes the results of the different methods according to these measures.

**Figure 5:**
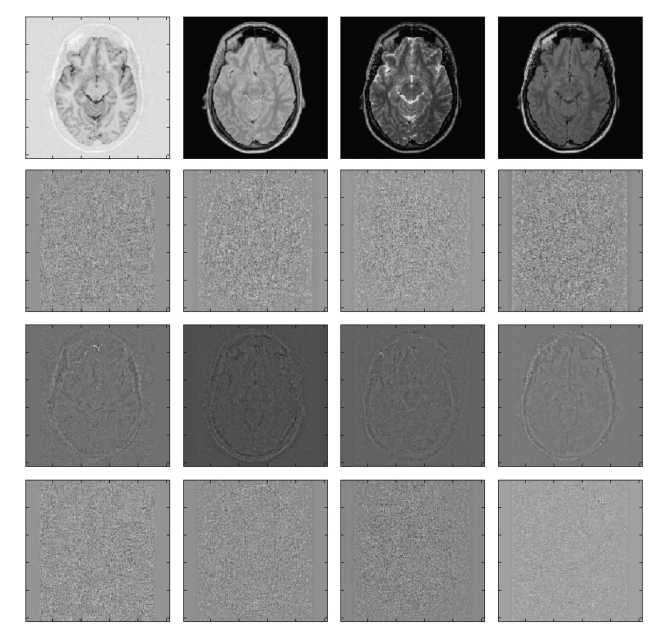
Denoising results (from top to down: reference original noisy images, residuals from OMNLM-PCA, MPVM, and MGMM resp.). As can be noticed, no appreciable anatomical structures are visible in the proposed method.

As can be observed, MPVM method tends to overcorrect the data, as the number of outliers is much higher than the expected for an optimal filter (this can be noticed in Figure 5 where image edges in the residuals are clearly visible). This is probably related to the modelling of each pure tissue as one grey-level value across the entire image. It has to be considered that the proportion of outliers measured for MPVM method represents an upper bound due to the removal of spatially correlated residuals introduced by field inhomogeneity artifacts. On the other hand, MGMM method performed well showing a good agreement with results obtained for the same level of noise (2%) on synthetic images. The OMNLM-PCA method performed the best according to both the measures. It removed the highest quantity of noise (77% in average) while showing a number of outliers very close from the theoretically expected for an optimum filter (60 in this case). Figures 4 and 5 show the filtered images with the compared methods and the corresponding residuals for visual comparison.

## 4. Conclusion/Discussion

In this paper, a new method for multicomponent MR image denoising has been proposed and evaluated using both synthetic and real clinical data. The proposed method has been compared with related state-of-the art methods.

 It has been demonstrated that using multicomponent images to denoising image series presents important benefits over single image techniques due to the increased data redundancy.

 Our proposed filter removes noise in the image domain by averaging similar patches around the image by using a robust multicomponent similarity measure which has been shown to improve the results of the MNLM method. Besides, the proposed filter also removes noise in the intercomponent domain by using a local PCA-based decomposition. The proposed method has shown a consistent improvement in the results when the number of images increases in contrast with the erratic behaviour of the basic version.

 The proposed method has been shown to outperform significantly all the other compared methods. The MPVM method showed a poor performance, especially at high noise levels. This can be attributed to a more cautious image formation model, which was designed to avoid the use of spatial smoothness constraints for some clinical applications. The MGMM method showed numerically a good performance but a significant blurring effect was present in the filtered images.

 There are a number of factors that can influence the denoising results of the proposed method such as image inhomogeneities, incorrect image registration or spatially dependent noise.

 As the denoising is performed using a local region surrounding the processed pixel the image inhomogeneities are expected to have a low impact in the final results. However, if an inhomogeneity correction is performed prior the denoising process, this effect can be minimized at the expense of spatially modulating the noise amplitude. A more significant factor is the correct registration of the image series. However, it has been observed that as the similarity measure is computed from several images mainly the incorrectly registered image(s) will be affected by this factor due to the robustness of the similarity measure. Besides, if registration errors are present, this will not cause an incorrect denoising but a suboptimal one since the preselection step only will reduce the number of similar patches as it is based on the intersection of distances of the local means.

 Finally, it has to be noticed that modern MRI is increasingly acquired using parallel imaging which leads to spatially-dependent and variable noise pattern. In such case the proposed method only requires an estimation of the local image noise and therefore an automated local noise estimator is required. In this sense, there are several works dealing with this issue [24, 25]. Other possibility is to obtain local noise estimations from the less significant local principal components [19]. These issues will be addressed with more research in near future.

 The implementation of the proposed method in the 3D case can potentially improve the results by increasing the number of similar pixels in the local surrounding volume and by using a more specific local similarity volume. In this work, we have chosen a 2D implementation to ease the comparison with other multicomponent state-of-the art methods. Besides, it has to be noticed that in 3D the execution time may be an issue for some implementations. However, as the proposed method is highly parallelizable the processing time associated with this technique can be dramatically reduced using distributed computing and related techniques [17].

 There are a number of possible applications of the proposed method to improve the quality of the images acquired on dynamic series such as fMRI, perfusion and diffusion weighted imaging or MR relaxometry. The application of the proposed methodology in these fields has to be addressed with further research.

## Figures and Tables

**Table 1:** Comparison of the MNLM and proposed filters for different noise levels. MRMSE from all the images is presented. MRMSE of the unprocessed images is presented for reference.

Noise Std. Dev. (%)	MRMSE			
	Noisy	MNLM	OMNLM	OMNLM-PCA
1%	2.37	2.22	1.42	1.09
3%	7.19	3.57	2.78	2.47
5%	11.97	5.58	3.81	3.57
7%	16.72	7.60	4.78	4.55
9%	21.52	9.71	5.95	5.79

**Table 2:** Comparison of the MNLM method and the proposed method for different number of images. RMSE in the T1 image is presented for the filtering with each number of components and noise level.

Noise Std. Dev. (%)	RMSE							
	MNLM				OMNLM-PCA			
	1	2	3	4	1	2	3	4
1%	2.03	2.02	2.00	2.00	1.65	1.22	1.13	1.10
3%	3.58	3.24	3.24	3.34	3.69	2.61	2.42	2.37
5%	5.55	5.02	5.09	5.47	5.58	3.81	3.53	3.45
7%	7.64	7.06	7.24	7.88	7.32	5.12	4.66	4.58
9%	9.30	8.54	8.88	9.60	9.34	6.35	5.79	5.66

Average	**5.62**	**5.18**	**5.29**	**5.66**	**5.51**	**3.82**	**3.51**	**3.43**

**Table 3:** Comparison of the proposed filter with MGMM and MPVM methods for different noise levels. The original MRMSE of the 4 unprocessed images was supplied for reference purposes. As can be noticed the proposed method was the best for all noise levels.

Noise Std. Dev. (%)	MRMSE			
	Noisy	MVPM	MGMM	OMNLM-PCA
1%	2.39	2.45	1.39	**1.09**
3%	7.18	3.27	3.22	**2.47**
5%	11.92	5.20	4.65	**3.57**
7%	16.75	7.79	5.90	**4.55**
9%	21.50	10.64	6.95	**5.79**

Average	11.95	5.87	4.42	**3.49**

**Table 4:** Comparison of the results of the different filters. RNF following filtering and data lying beyond 3 SD of original value following filtering (in brackets). The expected number of outliers was 60 for the selected brain region.

	Noise	MPVM	MGMM	OMNLM-PCA
IRTSE	58.76(2.1%)	0.77(292)	0.34(84)	**0.33 (51)**
PD	64.06(2.9%)	0.44(250)	0.30(68)	**0.23 (52)**
T2	58.20(3.3%)	0.43(174)	0.26(95)	**0.19 (74)**
FLAIR	52.40(2.2%)	0.39(246)	0.25(79)	**0.18 (69)**

Average	58.35(2.6%)	0.51(240)	0.28(81)	**0.23 (61)**

## Abbreviations

NLM: Nonlocal Means
MNLM: Multicomponent Nonlocal Means
OMNLM: Optimized Multicomponent Nonlocal Means
MPVM: Multidimensional Partial volume Modelling
MGMM: Multicomponent Gaussian Mixture Modelling
ADF: Anisotropic Diffusion Filter
PCA: Principal component Analysis
RGB: Red, Green, and Blue
MSE: Mean Squared Error
